# Climate-mediated diversification of turtles in the Cretaceous

**DOI:** 10.1038/ncomms8848

**Published:** 2015-08-03

**Authors:** David B. Nicholson, Patricia A. Holroyd, Roger B. J. Benson, Paul M. Barrett

**Affiliations:** 1 Department of Earth Sciences, The Natural History Museum, Cromwell Road, London, SW7 5BD, UK; 2 Museum of Paleontology, University of California, 1101 Valley Life Sciences Building, Berkeley, California 94720, USA; 3 Department of Earth Sciences, University of Oxford, South Parks Road, Oxford OX1 3AN, UK

## Abstract

Chelonians are ectothermic, with an extensive fossil record preserved in diverse palaeoenvironmental settings: consequently, they represent excellent models for investigating organismal response to long-term environmental change. We present the first Mesozoic chelonian taxic richness curve, subsampled to remove geological/collection biases, and demonstrate that their palaeolatitudinal distributions were climate mediated. At the Jurassic/Cretaceous transition, marine taxa exhibit minimal diversity change, whereas non-marine diversity increases. A Late Cretaceous peak in ‘global' non-marine subsampled richness coincides with high palaeolatitude occurrences and the Cretaceous thermal maximum (CTM): however, this peak also records increased geographic sampling and is not recovered in continental-scale diversity patterns. Nevertheless, a model-detrended richness series (insensitive to geographic sampling) also recovers a Late Cretaceous peak, suggesting genuine geographic range expansion among non-marine turtles during the CTM. Increased Late Cretaceous diversity derives from intensive North American sampling, but subsampling indicates that Early Cretaceous European/Asian diversity may have exceeded that of Late Cretaceous North America.

## Abstract (web summary)

Turtles are ectothermic vertebrates that have experienced major environmental perturbations. Here the authors show that the geographical distribution of turtles was mediated by climate throughout the Mezozoic and show an increase in diversity of non-marine turtles starting in the Early Cretaceous.

## Graphical abstract

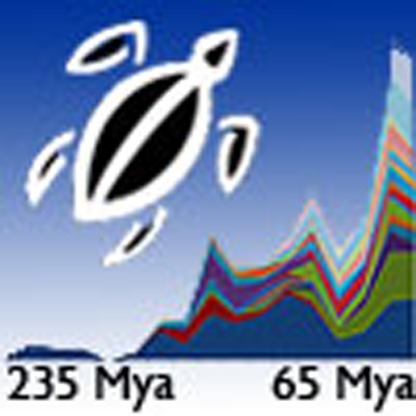

Turtles, tortoises and terrapins (collectively chelonians) are successful vertebrates that have endured for over 220 million years and have persisted through several major environmental perturbations, including the formation of global hothouse conditions in the Late Cretaceous and Eocene (and associated cooling events), the end-Cretaceous mass extinction and post-Eocene global cooling trends. Chelonians inhabit marine, freshwater and terrestrial environments, and are abundant as fossils, have a well-established taxonomy and their habitat preferences are well-understood, making them a model system for studying the responses of ectothermic vertebrates to long-term environmental change. However, few studies have attempted to document long-term trends in chelonian palaeobiodiversity and whether any of these trends in species-richness might represent genuine evolutionary or biotic events, responses to environmental change or whether they may simply be artefactual results of sampling or geological biases.

The earliest known chelonians first appeared in the Late Triassic within a few million years of the earliest dinosaurs (for example, refs 1, 2, 3, 4) and achieved a global distribution shortly thereafter, evolving to take advantage of both aquatic (freshwater and marine) and terrestrial habitats^5^. In addition to the invasion of new ecological niches and geographical areas, a growing body of evidence is pushing the origins of many extant chelonian clades into the Early Cretaceous^6^,^7^, suggesting that chelonians underwent as rapid and profound an adaptive radiation in the Mesozoic as many other vertebrate groups. In contrast to most other Mesozoic vertebrates, chelonian lineages were little affected by the K/Pg extinction event^8^,^9^: thus we can trace the origins of modern families into the Cretaceous^10^,^11^, finding recognizable relatives of animals that are today endangered by habitat loss, climate change and predation^12^,^13^ and that are commonly used in evolutionary and developmental studies^14^,^15^,^16^,^17^,^18^. Of the ∼317–323 accepted extant chelonian species, an estimated 51.2–63% are threatened—a remarkably high proportion, even when compared with other at-risk ectotherms^12^,^13^.

While the Mesozoic diversifications of mammals, dinosaurs and other tetrapod clades have received considerable attention (for example, refs 19, 20, 21, 22), the equally remarkable diversification of chelonians has not been explored in detail nor at a global scale. Previous studies focused on taxonomic treatments of selected clades^23^,^24^,^25^,^26^,^27^,^28^ or turtle faunas on restrictive local- to continental-level scales^29^,^30^,^31^. Only a handful of studies have explicitly quantified this taxonomic diversity on even regional scales and these have concentrated only on selected regions of western North America in the Late Cretaceous^9^,^32^,^33^. Here we present the first comprehensive and quantitative analysis of chelonian palaeobiodiversity through the Mesozoic to understand the temporal and spatial patterns of turtle diversification on a global scale, examining their evolution in the context of global climate change and establishing the general pattern and timing of their diversification in relation to the radiations of other co-occurring tetrapods.

The distribution of Mesozoic chelonian fossil occurrences indicates climate-mediated geographical distributions, with chelonians absent from high palaeolatitude tetrapod localities. At the Jurassic/Cretaceous transition, marine chelonians show no substantive change in richness while global non-marine chelonians were in the initial stages of a long-term Early Cretaceous diversity increase. A Late Cretaceous (Turonian) peak in global non-marine subsampled richness coincides with high palaeolatitude occurrences and the Cretaceous thermal maximum (CTM). This apparent Late Cretaceous ‘global' diversity increase results from expansion of the number of well-sampled geographic regions, and is not corroborated by diversity patterns within regions such as North America, Asia or Europe. However, an alternative, model-detrended richness series, which is not sensitive to fluctuations in geographic sampling, supports this peak, suggesting that this high richness was achieved by a genuine expansion of the geographic range of turtles on land during the warmest interval of the past 250 million years. The apparent steep increase in raw chelonian diversity during the Late Cretaceous is a function of relatively complete sampling in North America, while subsampling indicates that genus richness in the Early Cretaceous of Europe and Asia may have exceeded that seen in Late Cretaceous North America.

## Results

### Palaeolatitudinal distribution of fossils

**Figure 1:**
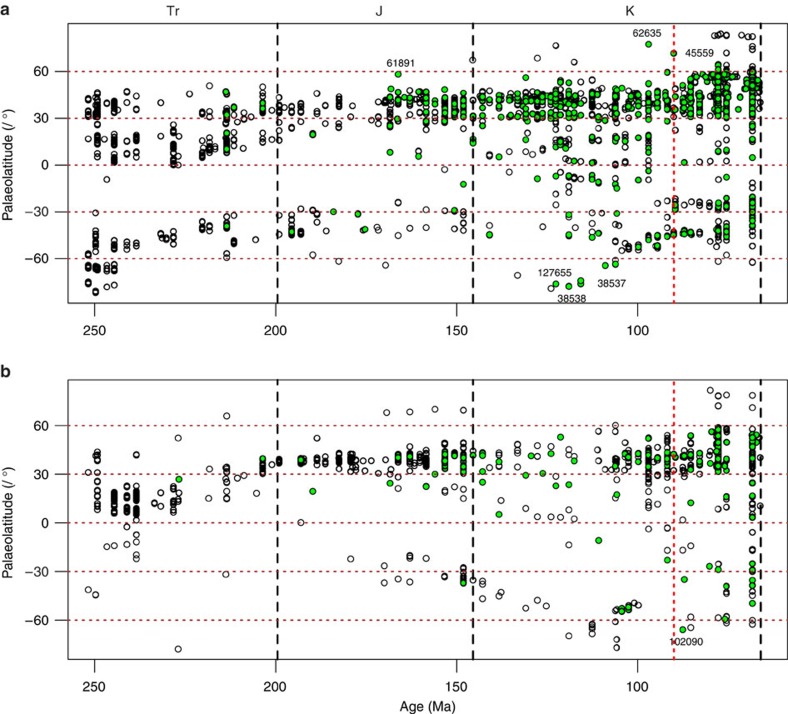
Palaeolatitudinal distribution of chelonians and other tetrapods through the Mesozoic. (**a**) Non-marine and (**b**) marine: open circles—tetrapod occurrences; green circles—chelonian occurrences. J, Jurassic; K, Cretaceous; Tr, Triassic. Circles identified with numbers represent specific PBDB collections mentioned in the text.

The palaeolatitudinal distribution of marine and non-marine chelonians is broadly similar to that of other Mesozoic tetrapods but showing a truncated distribution at high latitudes, with the greatest concentrations of occurrences between 30 and 60° N (Fig. 1). Notable excursions in the non-marine data (Fig. 1a) into northern high latitudes occur in the Middle Jurassic (PBDB collection (coll.) 61891: *Xinjiangchelys* sp. from the Itat Formation, Krasnoyarsk, Russia^34^, 58.17° N, Bathonian) and Upper Cretaceous (PBDB coll. 62635: indeterminate material from the Chandler Formation, Alaska^35^, 77.42° N, Cenomanian, originally suggested to be a dermatemydid by comparison with taxa which are now placed in Lindholmemydidae; and PBDB coll. 45559: *Borealochelys*, the macrobaenid *Aurorachelys* and a generically indeterminate trionychid from the Kanguk Formation, Nunavut, Canada^36^, 71.62° N, Turonian–Coniacian). Notable excursions into southern high latitudes include a cluster of collections from the Lower Cretaceous of Victoria, Australia^37^,^38^,^39^, ∼76–78° S (for example, PBDB colls. 127655, 38538, 38537, 38534: *Otwayemys* plus indeterminate material, Aptian-Albian). The latest Cretaceous presence of other tetrapod clades in the far-northerly latitudes (particularly from Canada and the North Slope of Alaska) is not matched by a presence of chelonians, despite broadly tracking tetrapod distribution throughout the Mesozoic.

### Correlations between genus counts and collections

Chelonian genus counts through the Mesozoic are strongly correlated with the number of tetrapod-bearing collections. This relationship is strongest for the marine subset (Spearman's **ρ**=0.795) but is still strongly related in the much larger non-marine subset (**ρ**=0.673).

### Global genus counts and subsampled richness

**Figure 2:**
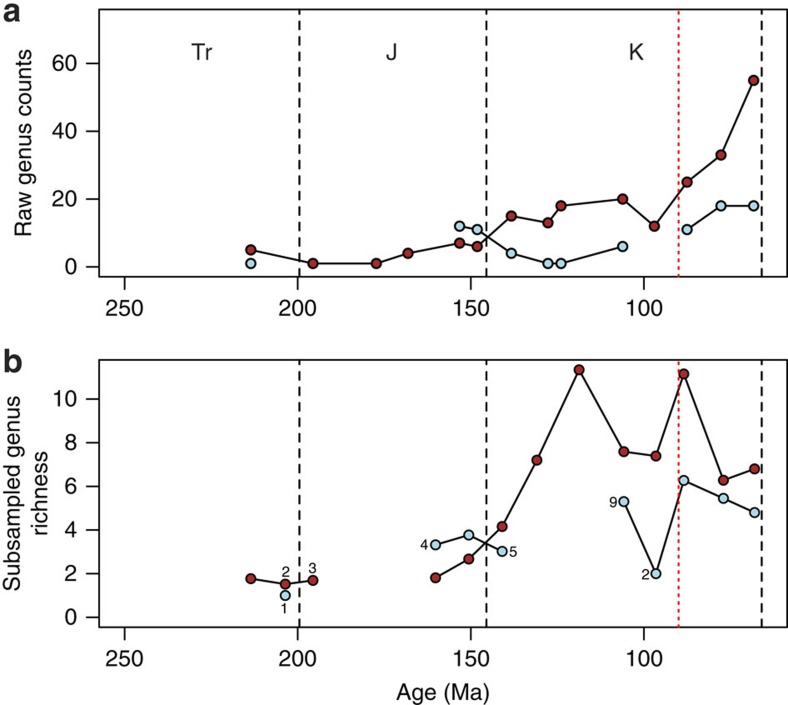
Global genus richness of Mesozoic chelonians. (**a**) Raw counts and (**b**) after Shareholder Quorum Subsampling. Light blue circles=marine taxa; brown circles=non-marine taxa. Data points drawn from a sampling pool derived from fewer than 10 publications are marked with the number of publications. J, Jurassic; K, Cretaceous; Tr, Triassic.

Raw counts of globally sampled-in-bin, non-marine turtle genera (Fig. 2a) show a very gradual increase in richness through the Jurassic and a marked increase in the lowermost Cretaceous. Continued, gradual increases in these counts during the mid-Cretaceous are punctuated by a sharp decline in the Cenomanian, followed by a steep increase to the highest counts during the Maastrichtian. By contrast, marine taxa are absent from the fossil record after their initial appearance in the Late Triassic^3^ but reappear with higher counts than the non-marine taxa in the Kimmeridgian-Tithonian representing the evolution of nearshore marine habits in groups such as Plesiochelyidae (for example, ref. 40). Marine turtle genus counts then exhibit a Lower Cretaceous decline, after the final appearances of ‘Jurassic' marine clades in Europe^17^, and subsequent appearance of bothremydids (extinct pleurodires inhabiting brackish and marine environments) and chelonioids (the extant clade of marine turtles) in the Aptian-Albian of Brazil^25^ and the UK^41^,^42^. An absence of definite marine turtle occurrences from the fossil record of the early Late Cretaceous (due in part to poor dating resolution of occurrences which may fall in this interval) is concurrent with the Cenomanian decrease in non-marine turtle genus counts, and a less rapid increase to a latest Cretaceous richness that is only marginally higher than that seen in the Upper Jurassic.

The Shareholder Quorum Subsampling (SQS) subsampled curve preserves some features of the raw taxonomic curves, but contrasts strongly in others (Fig. 2b; Supplementary Figs 1 and 2). The concurrent increase in terrestrial diversity and decrease in marine diversity over the Jurassic-Cretaceous boundary (Fig. 2a) is modified in the SQS curves. The subsampled decrease in marine diversity is very slight and should be treated cautiously as earliest Cretaceous marine turtles have only been reported or restudied in a small number of publications (five; Fig. 2b). In the terrestrial SQS curve, the greatest increase in richness is seen in the first three Cretaceous time-bins (compared with the latest Cretaceous increase in Fig. 2a). The sharp Cenomanian drop in directly counted global genera in both the marine and terrestrial realms is reproduced in the SQS curves to some extent, but the subsequent large increase in observed diversity becomes a general decrease when subsampled. In the marine data, the absence of occurrences during the Cenomanian translates to a large drop in diversity in the SQS curve (due to the SQS binning protocol above, where occurrences spanning multiple time-bins are assigned to that which makes up more than half, while the observed curve is made up only of those records which fall discretely into the time-bins; but also note that the Cenomanian marine data is not robust to issues of data quality due to being drawn from only two publications; Fig. 2b) and a gradual decrease in richness is recovered for both terrestrial and marine taxa towards the end of the Cretaceous.

### Regional non-marine genus counts and subsampled richness

**Figure 3:**
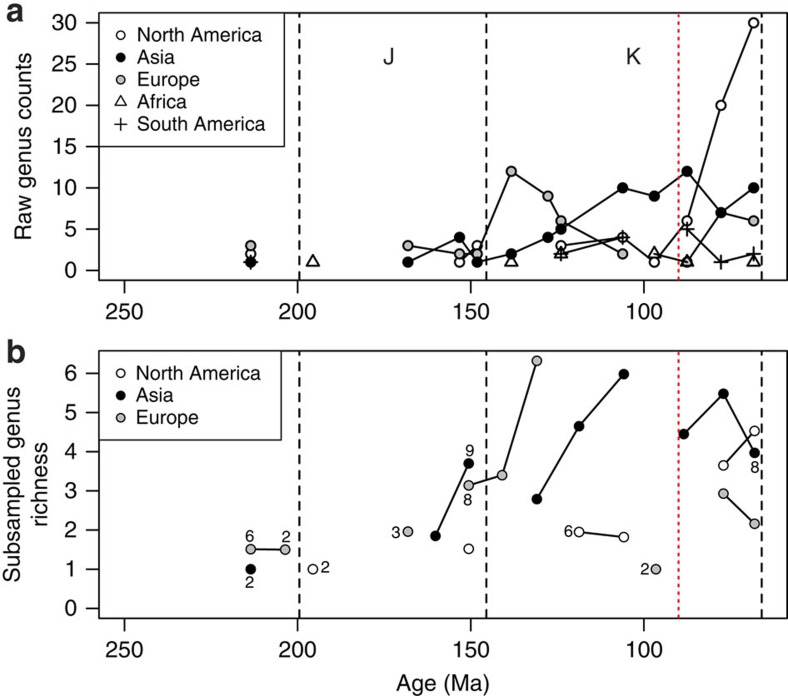
Genus richness of non-marine chelonians by continent. (**a**) Raw counts and (**b**) after Shareholder Quorum Subsampling. Data points drawn from a sampling pool derived from fewer than 10 publications are marked with the number of publications. J, Jurassic; K, Cretaceous. Subsampled richness for Africa and South America not shown.

Non-marine faunas are highly regionalized compared with marine faunas, with very few genera shared between continents. This means that ‘global' patterns, even when applying subsampling, can be biased by differences in the palaeogeographical spread of localities from different intervals and should not be read literally as patterns of standing diversity. Therefore, in addition to the ‘global' results reported above, we analysed non-marine turtle diversity by geographical regions (Fig. 3a; Supplementary Table 1). The observed regional genus counts show geographical heterogeneity in non-marine diversity through time (Australia has only three non-marine genera with a total of seven occurrences, so is not shown here). The ‘global' increase in genus counts seen across the Jurassic-Cretaceous boundary (Fig. 2) is driven largely by high European counts (Fig. 3a). A subsequent European decrease in Lower Cretaceous time-bins contrasts with an increase in the observed genus counts of the Asian fauna during the same interval. Data from North America only form a substantive part of the data set in the final three Cretaceous time-bins (Fig. 3a), where they drive the majority of the rapid increase in non-marine diversity seen in the raw global genus counts (Fig. 2a). Africa and South America contribute little to the global record of Mesozoic turtles, although relative patterns of observed South American counts follow a similar trajectory to those of Asia, but at lower observed genus counts (Fig. 3a).

The SQS analysis of the regional data replicates some features of the raw observed data but, as with the ‘global' data, contrasts with others (Fig. 3b). Subsampled non-marine turtle richness was low during the Late Triassic and remained so in most Jurassic regions and time-bins. Turtles are known from relatively few Triassic-Middle Jurassic localities, raising the possibility that this low diversity signal results from low sample quality (Fig. 3b). However, it is more likely that it reflects the genuine scarcity of the clade during its early history, because fossil record sampling of this protracted interval has been intensive^43^. The subsampled regional data show that the Cretaceous diversity of non-marine turtles generally exceeded that of the Late Triassic and Jurassic, a pattern that is presaged by elevated diversity in the latest Jurassic of Europe and Asia (Fig. 3b). Substantial regional variation in Cretaceous turtle diversity might be related to historical biogeographical patterns or the distribution of facies and facies diversity. The sharp increase in European turtle genus counts across the Jurassic-Cretaceous boundary is essentially absent from the SQS curve, although a substantial increase in subsampled diversity did occur between the Berriasian/Valanginian (earliest Cretaceous) and Hauterivian/Barremian (mid-Early Cretaceous). The timing of the subsequent decline is unresolved as too few data are available for the subsampling protocol (even at lower quorum levels; Supplementary Figs 3–5). However, the subsampled richness of European turtle genera had decreased by the final Cretaceous bins (Campanian-Maastrichtian). In the Asian curves, most features of the raw genus counts are reproduced by the SQS subsampled richness estimates with the exception of those in the final three latest Cretaceous bins, where an observed reduction before an increase to the value in the final time-bin in the raw taxic curve is reversed in the SQS curve. In the North American curves, the richness increase at the end of the Cretaceous is retained by SQS, although not to the highest genus-richness levels seen overall, as Early Cretaceous peaks in both European and Asian subsampled richness are higher than those recovered for the latest Cretaceous of North America (Fig. 3b).

### Residuals analysis

Model residuals of both non-marine and marine taxa (Supplementary Figs 6 and 7; Supplementary Table 2) show a general pattern of expected or lower than expected richness in the Jurassic and expected or higher than expected in the Cretaceous, consistent with the broad pattern in the SQS analyses indicating higher richness in the Cretaceous than Jurassic. The strong negative excursion in the final Jurassic bin in both sets does not match the pattern in the SQS global curves (Fig. 2b), however, the SQS diversity peak in non-marine turtles in Cretaceous bin 3 (Fig. 2b) is replicated by a strong positive excursion in that bin in the residuals analysis (Supplementary Fig. 6). Most importantly for the conclusions of this study, the apparent SQS global non-marine diversity peak in Cretaceous bin 6 (Fig. 2b), coinciding with the CTM but not seen in any individual well-sampled geographic area (Fig. 3b), is supported by a positive peak in the residuals analysis (Supplementary Fig. 6).

## Discussion

Strong correlations between chelonian taxic richness and the number of tetrapod-bearing PBDB colls. indicate that some features of the raw genus count curves (Figs 2a and 3a) may be influenced by sampling biases in the fossil record, justifying the use of a subsampling protocol to remove these distortions^44^.

In the marine realm, observed chelonian genus counts suggest a slight reduction in richness across the Jurassic-Cretaceous boundary (Fig. 2a). However, the decline in the SQS curve is negligible and the low numbers of publications for earliest Cretaceous marine turtles (Fig. 2b) suggest that low uncorrected genus counts may be a result of excessively poor sampling in that time bin. A gap in the marine SQS curve coincides with the disappearance of the marine basal eucryptodires (which radiated in the Late Jurassic) and the subsequent appearance of the more derived Chelonioidea in the Cretaceous^17^. This turnover coincides with a mid-Early Cretaceous regression and taxonomic turnovers among other shallow marine reptile groups^21^,^45^, with other classically ‘Jurassic' marine tetrapod clades generally extending into the earliest Cretaceous^46^. Contrasting with this is a coincident increase in non-marine chelonian richness, which is seen in both the raw data and the SQS analysis. This indicates that chelonians in the marine and non-marine realms experienced different availability of ecological opportunities, potentially driven by changes in habitat distribution and perhaps related to a fall in global eustatic sea level at this time^47^ that reduced shallow marine shelf area, or that the evolution of key innovations opened new ecological opportunities for non-marine turtles. The global Cretaceous non-marine curves for the observed and subsampled data differ considerably (Fig. 2) with the largest richness recovered as either Early (subsampled) or Late Cretaceous (observed). Subdivision of these data by continent (Fig. 3) shows that this temporal shift in the timing of diversification between raw and subsampled data is likely a function of relative greater raw sampling of Late Cretaceous North American fossils compared with those from Early Cretaceous European and Asian faunas, suggesting that the potential for discovering new genera of chelonians from Europe or Asia is much greater than in North America, where sampling is relatively complete. The broad pattern in the regional analyses shows globally low richness in the Triassic and much of the Jurassic, with regional increases in Europe and Asia during the Late Jurassic. Cretaceous subsampled richness varied prominently by interval and region, in many cases attaining higher values than those seen previously, for instance in the earliest Cretaceous of Europe, the late Early Cretaceous onwards in Asia and the Campanian-Maastrichtian of North America. Overall, these patterns suggest that a significant episode of turtle diversification began in the Late Jurassic and continued into the Cretaceous, resulting in a geographically heterogeneous diversity profile. Environmental, climatic or habitat differences might have caused this heterogeneity, a possibility that will be tested by future work.

Subsampled marine and non-marine data suggest that a strong increase in raw genus counts in the final three time-bins of the Cretaceous is likely a sampling artefact, and that richness generally decreased across this interval. It is interesting to note that the last peak in subsampled richness for both marine and non-marine taxa in Cretaceous bin 6 (Turonian-Santonian) coincides with the CTM^48^, which is potentially significant given the ectothermic physiology of chelonians and the possible role of temperature in driving modern distributions^49^. However, as SQS (and other subsampling methods) are intended to accurately estimate the relative size of the taxon pool from which occurrences are drawn, it is sensitive to changes in geographic area that such a ‘global' data set is biased by differences in the palaeogeographical spread of localities among intervals. Confirming this, the apparently high ‘global' subsampled richness of Cretaceous bin 6 is not reflected in any one of the well-sampled regions using SQS (Fig. 3b) or classical rarefaction (CR; Supplementary Figs 8 and 9), and is therefore probably the result of inclusion of better sampling in additional areas (that is, South America) in this bin than at other times in the series (Fig. 3a). While ‘global' subsampling curves cannot distinguish genuine range contraction/expansions from reduction/expansion in the geographic spread of fossil record sampling, a global residual-based approach using collections from which any non-marine tetrapods have been found as a sampling proxy includes a component of geographic spread. Therefore, the recovery of a peak in global non-marine residual diversity at Cretaceous bin 6 (Supplementary Fig. 6) may represent genuinely high global diversity, and the absence of this peak from the regional subsampling curves (Fig. 3b) suggests that this was achieved by a genuine expansion of the geographic range of turtles on land during the warmest interval of the past 250 million years. Furthermore, the Early Cretaceous increase in richness captured in the SQS curves matches the timing of modern chelonian lineage splits that have been identified in molecular phylogenies^6^,^50^, suggesting that the SQS curves are accurately identifying periods of high taxic richness.

**Figure 4:**
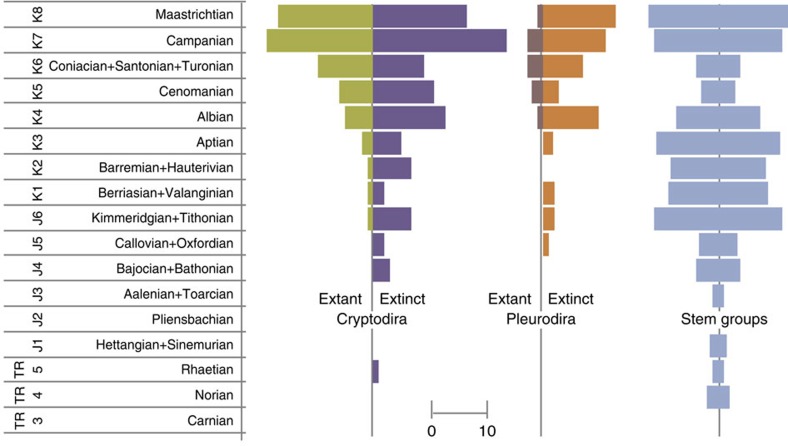
Chelonian genera through time by suborder. Spindle diagram showing raw counts of genera through time divided into the suborders Cryptodira, Pleurodira and other stem groups.

Although molecular and palaeontological data generally concur on the timing of origin of modern turtle lineages in the Early to ‘middle' Cretaceous (for example, refs 6, 7, 11), few of these lineages attained substantial diversity during the Mesozoic. Trionychidae is an exception, however, and becomes one of the most diverse family-level clades by the end of the Cretaceous and common-to-dominant in many assemblages^9^,^32^. Regarding the timing and diversification of different clades from known fossils, when viewed as proportions of genera observed in each interval, no clear signal of family-level replacement emerges as a potential cause of richness change through time (Supplementary Figs 10–12; Supplementary Table 3). However, proportions of observed genera representing the suborders Cryptodira and Pleurodira show that changes in diversity are being driven by the Cretaceous diversification of modern cryptodiran lineages^23^, while pleurodires contribute comparatively little to changes in taxic richness (Fig. 4).

Chelonian palaeolatitudinal distribution through time broadly follows that for all other tetrapods in both marine and non-marine environments (Fig. 1), but chelonians are almost always absent from latitudes above 60° N and 60° S, whereas other tetrapods are well-represented. This discordance indicates that additional environmental conditions, presumably temperature or precipitation related, were preventing colonization by and/or the persistence of turtles at high palaeolatitudes through most of the Mesozoic. As with the Late Cretaceous peak in subsampled richness (Fig. 2b), the only collection with appreciable chelonian richness above 60° N (PBDB coll. 45559/Nunavut) coincides with the CTM. This observation is consistent with the climatically limited distribution of crocodilians in the Mesozoic and Cenozoic^51^. However, interpretations of chelonian distribution should be cautious, due to the varying temperature tolerances among turtle taxa (some can hibernate or aestivate), the complex interplay of temperature and precipitation on their Recent distributions^49^, and the influence of photoperiodicity and temperature on the reproductive cycles of turtles and other reptiles^52^,^53^. Further research on the climatic tolerances of modern and extinct chelonian lineages will help to elucidate the relative importance of climate in the past or if, in the more equable climate of the Mesozoic, available land area is a better explanation for their distribution, as recently demonstrated for dinosaurs^54^.

## Methods

### Nomenclatural note

For the benefit of British readers, ‘turtles' in the title is used in the broadest sense, commonly employed in North America, to include all Testudinata; that is, the total group comprising all crown chelonians (turtles, terrapins and tortoises) and the extinct lineages in their stem. ‘Turtle' in the more restrictive and informal sense (not including terrapins and tortoises) is a paraphyletic grouping of freshwater and marine taxa and so the more inclusive usage is preferred.

### Data download

Mesozoic chelonian occurrences (Supplementary Data 1) were downloaded from the Paleobiology Database^55^ (PBDB: www.paleobiodb.org) through Fossilworks (www.fossilworks.org), following a comprehensive literature search and databasing effort by two of the authors of this paper (D.B.N. and R.B.J.B.), adding to the considerable earlier efforts of Matthew Carrano (52.7%), John Alroy (12.9%) and Roger Benson (9.1%) with additional contributions by Martin Aberhan, Anna Behrensmeyer, Kevin Boyce, Richard Butler, Matthew Clapham, Emmanuel Fara, Jason Head, Austin Hendy, Pat Holroyd, Linda Ivany, Wolfgang Kiessling, Matt Kosnik, Conrad Labandeira, Graeme Lloyd, Philip Mannion, David Nicholson, Mark Patzkowsky, Shanan Peters, Oliver Rauhut, Allister Rees, Mark Uhen, Loic Villier and Robin Whatley. The data were downloaded using the search terms ‘Testudinata' and ‘Mesozoic' for the taxonomic and stratigraphic fields, respectively. Occurrences were downloaded at species level and inclusive of generically indeterminate material. At the time of download (14 September 2014), this comprised 3,086 occurrences in 1,599 collections and, within this, 1,705 occurrences in 1,012 PBDB colls. with genus-level identifications. A second data set (Supplementary Data 2) of all Mesozoic tetrapods was downloaded^56^ (31 July 2014), using the search terms ‘Tetrapoda' and ‘Mesozoic', to compare the palaeolatitudinal distribution of chelonian occurrences with those of other tetrapods. The major contributors to the data set are Matthew Carrano (51.9%), Richard Butler (13.2%), John Alroy (9.3%), Philip Mannion (7.5%) and Roger Benson (6.8%), with additional contributions by Martin Aberhan, Anna Behrensmeyer, Mark Bell, David Bottjer, Kevin Boyce, Matthew Clapham, Will Clyde, Emmanuel Fara, Jason Head, Austin Hendy, Pat Holroyd, John Hunter, Linda Ivany, Kirk Johnson, Wolfgang Kiessling, Matt Kosnik, Juergen Kriwet, Evelyn Kustatscher, Conrad Labandeira, Graeme Lloyd, Rick Lupia, Alistair McGowan, Johannes Mueller, David Nicholson, Robin O'Keefe, Mark Patzkowsky, Shanan Peters, Oliver Rauhut, Allister Rees, Ray Rogers, Chris Sidor, Dena Smith, Bruce Tiffney, Takehisa Tsubamoto, Mark Uhen, Matthew Vavrek, Loic Villier, Pete Wagner, Xiaoming Wang, Robin Whatley and Scott Wing.

### Correlations

To test the relationships between sampling and apparent diversity, correlations were performed between chelonian genus counts and numbers of tetrapod-bearing collections, as recorded in the PBDB after re-binning of the data for the analyses (see below), separately for both marine and non-marine occurrences of chelonians and all tetrapods.

### Palaeolatitude estimates and data subsetting

Due to the very different ecological influences and preservation modes in the marine and non-marine realms, occurrence data were subsetted according to the taxon's living environment. Where it was not possible to ascertain this by taxonomic information alone (for example, an occurrence labelled only ‘Cryptodira indet.'), the depositional environment was taken as a proxy for the living habitat. Although miscategorizations likely occur (and are more likely in marine deposits than non-marine), we believe this constitutes a reasonable effort to categorize these data for the purposes of investigating the geographic distribution of turtles.

The non-marine set was further subdivided by continent. Modern geopolitical boundaries are not an adequate marker for palaeogeographical classification in some cases, so occurrences by country were divided by geographic area as shown in Supplementary Table 1. India and Madagascar have very few occurrences in the data set (6 and 10, respectively) and can be considered separate continental masses, so were left out of the continent-level analysis. The geographical distribution of turtles through time was investigated by plotting the palaeolatitudes of all turtle occurrences (including generically indeterminate records) alongside other tetrapod occurrences, with the data separated into that derived from the marine and non-marine (terrestrial plus freshwater) realms. Palaeolatitude estimates used for the occurrences in Fig. 1 were generated by the Fossilworks website from the present-day coordinates of the collection, based on tectonic plate rotations from the PALEOMAP Project (www.scotese.com).

For the SQS analyses (see below), standard stratigraphic stages (as defined by the International Commission on Stratigraphy) were strategically merged in an attempt to even out the lengths of these intervals and to reduce the variance in their durations^57^ as follows: Tr3 (Carnian), Tr4 (Norian), Tr5 (Rhaetian), J1 (Hettangian, Sinemurian), J2 (Pliensbachian), J3 (Aalenian, Toarcian), J4 (Bajocian, Bathonian), J5 (Callovian, Oxfordian), J6 (Kimmeridgian, Tithonian), K1 (Berriasian, Valanginian), K2 (Barremian, Hauterivian), K3 (Aptian), K4 (Albian), K5 (Cenomanian), K6 (Coniacian, Santonian, Turonian), K7 (Campanian) and K8 (Maastrichtian). Presentation of the raw data can be found in Figs 2a and 3a. Data were binned using PBDB ‘10 million year' bins, as set out by Alroy *et al*.^58^, and data were only included if the occurrence fit into the bin (that is, the age range given did not cross bin boundaries).

### Subsampling

Recent studies have demonstrated that simplistic literal readings of the fossil record can lead to misleading palaeobiodiversity estimates due to biases introduced through variable preservation potential, geological megabiases and sampling intensity (for example, refs 19, 44, 59, 60, 61, 62, 63, but see refs 64, 65). Two major approaches for correcting these biases have been developed: modelling approaches that attempt to compare predicted richness measures based on variations in sampling or geological proxies through time with empirically derived data sets^66^,^67^ and more commonly used subsampling methods (for example, ref. 44). In our opinion, the most robust and fair subsampling protocol devised to date is SQS^44^, which samples to a pre-specified level of coverage (the ‘quorum') of the occurrence frequency distribution rather than imposing uniform sampling, thus avoiding the dampening of genuine fluctuations in diversity that characterize analyses using CR^44^,^68^. Empirical studies show that a quorum level of 0.4 is sufficient to accurately resolve changes in relative standing diversity^68^ and this level is presented in our main text figures, with results at different quorum levels shown in Supplementary Figs 1–5. We used the SQS Perl script version 4.3 (available from John Alroy on request) to analyse non-marine chelonian genus diversity at global and continental scales, where possible, based on the continents that contributed most to the global richness signal (North America, Europe and Asia), to gain insights into the influence of geographical sampling on the global diversity signal. Each subset of data was analysed at quorum levels from 0.1–0.8, with 1,000 trials per run. Default settings were used for most options with the following exceptions. Number of collections drawn per reference was set to three, and collections spanning multiple time-bins are assigned to whichever bin comprises more than half of the total age range. If no bin comprises more than half of the range then the occurrence is dropped from the analysis. We feel this is a very reasonable compromise between keeping as much data as possible, while removing those records with the worst stratigraphic resolution.

An indication of data quality for each time bin is given in the SQS plots by marking those points that sample from a pool comprising <10 publications with the relevant number of publications.

### Residuals and CR analyses

To complement the SQS analyses, a residuals analysis (a model-based approach, where observed diversity is subtracted from the expected diversity based on a sampling proxy^66^,^67^) and subsampled diversity based on CR were performed. We prefer SQS to CR, as CR attempts to correct for biases by imposing uniform sampling on all intervals, thus enforcing a low resolution of sampling by using the poorest observation level included in the data set across all intervals^44^. This has the undesirable effect of dampening genuine fluctuations in diversity by flattening the overall curve. Alternatively, SQS simulates a fair (rather than uniform) sample of true diversity by subsampling to a pre-specified level of coverage^44^,^68^. However, it should be noted that there is no perfect method for subsampling fossil occurrence data, and the best method will vary depending on the properties of the data set being analysed^69^. Hannisdal *et al*.^70^ performed sensitivity analyses with an older version of SQS (R script version 3.3, which does not perform many of the functions of the Perl script version 4.3 used here) under a variety of simulated sampling and data removal regimes, showing that SQS works very well under some of these, while it performs badly at estimating richness of the simulated taxon pool under others. Given these concerns, we feel it is appropriate to present the findings using multiple methods, as recommended by Bush *et al*.^69^

For the residuals analysis, we used the method and code provided by Lloyd^67^ (which is itself an extension and refinement of Smith and McGowan^66^) to model the expected diversity of Mesozoic marine and non-marine chelonians based on the number of tetrapod-bearing PBDB colls. per time bin. First, the fossil data and sampling proxy are independently sorted from lowest to highest values. A statistical model is then fitted to these sorted data to predict the expected values of the fossil data given a value of the relevant proxy. Smith and McGowan^66^ fitted only a linear model to the data (after log transformation) but Lloyd^67^ added logarithmic, exponential, sigmoidal and polynomial models to take account of any nonlinearity in the relationship between the variables. The best model is chosen by use of the sample size-corrected Akaike Information Criterion (AIC_c_). The predicted (modelled) values of richness are subtracted from the observed values and the residuals plotted. S.e. (1.96) and s.d. of the model are used as confidence intervals, whereby any point outside these limits represent a significant deviation from the expected (modelled) value.

We include these analyses as, unlike SQS, the use of a sampling proxy implicitly includes some measure of geographic area and so is not as sensitive to fluctuations resulting in the sporadic sampling of additional regions in ‘global' data sets.

## Additional information

**How to cite this article:** Nicholson, D. B. *et al*. Climate-mediated diversification of turtles in the Cretaceous. *Nat. Commun*. 6:7848 doi: 10.1038/ncomms8848 (2015).

## Supplementary Material

Supplementary Figures and Tables Supplementary Figures 1-12 and Supplementary Tables 1-3

Supplementary Data 1 Mesozoic Testudinata occurrences downloaded from www.Fossilworks.org on 2014-10-14

Supplementary Data 2 Tetrapoda occurrences downloaded from www.Fossilworks.org on 2014-07-31
