# Supplementary material for: Climate-mediated diversification of turtles in the Cretaceous
Source: Nat Commun. 2015 Aug 3;6:7848. doi: 10.1038/ncomms8848 (PMC4532850; doi:10.1038/ncomms8848)
Supplement: Supplementary Figures and Tables — Supplementary Figures 1-12 and Supplementary Tables 1-3 [file ncomms8848-s1.pdf]

Supplementary Figure 1

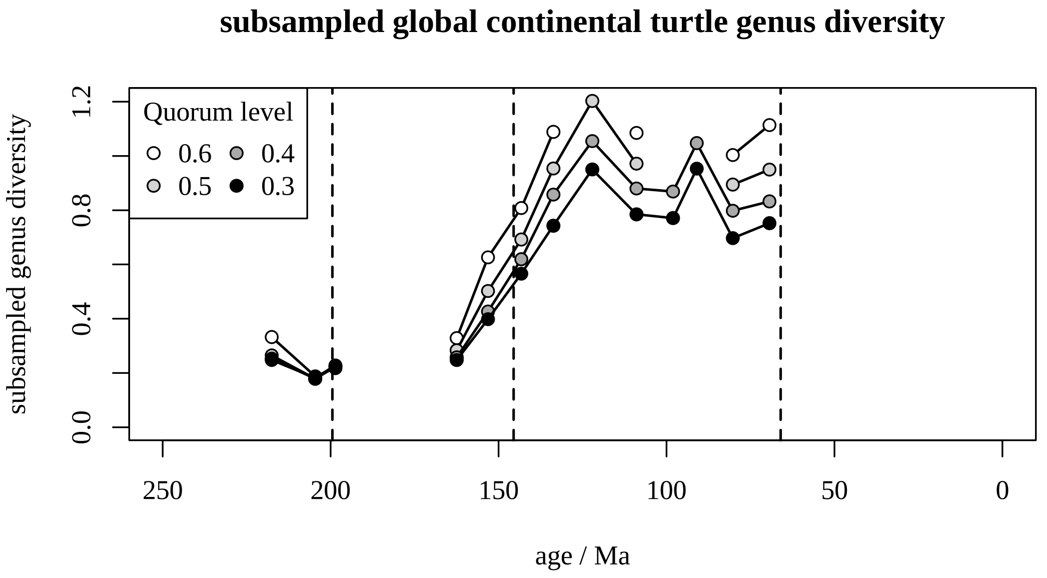

Subsampled global non-marine chelonian genus diversity
Data shown after log transformation, at four quorum levels.

Supplementary Figure 2

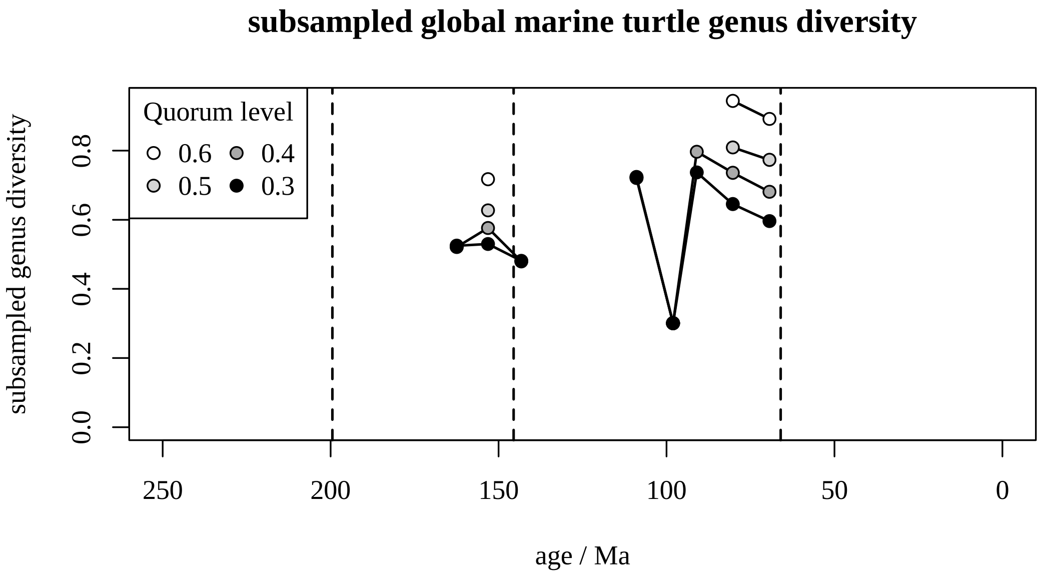

Subsampled global marine chelonian genus diversity
Data shown after log transformation, at four quorum levels.

Supplementary Figure 3

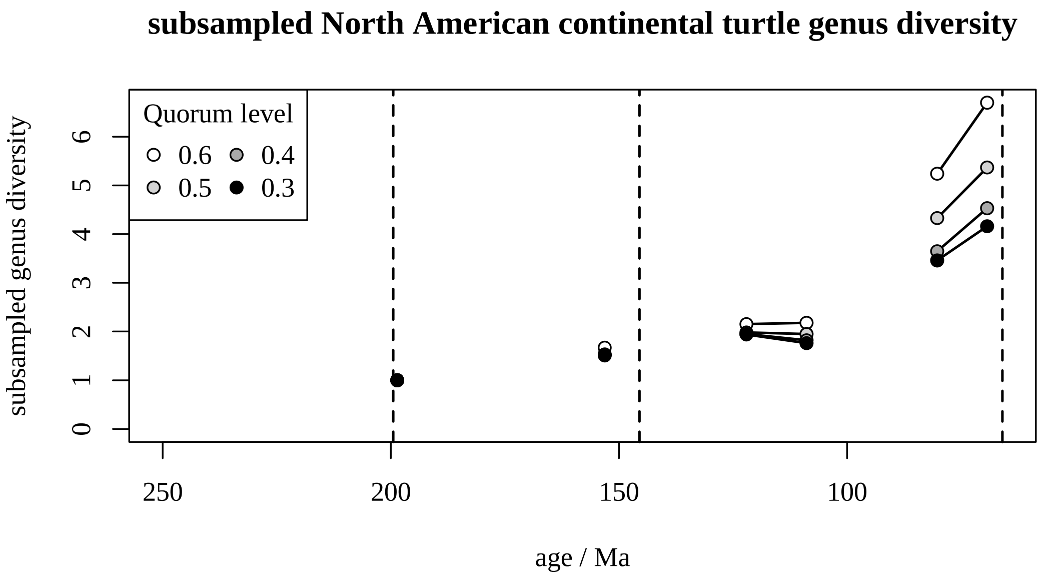

Subsampled North American non-marine chelonian genus diversity
Data shown at four quorum levels.

Supplementary Figure 4

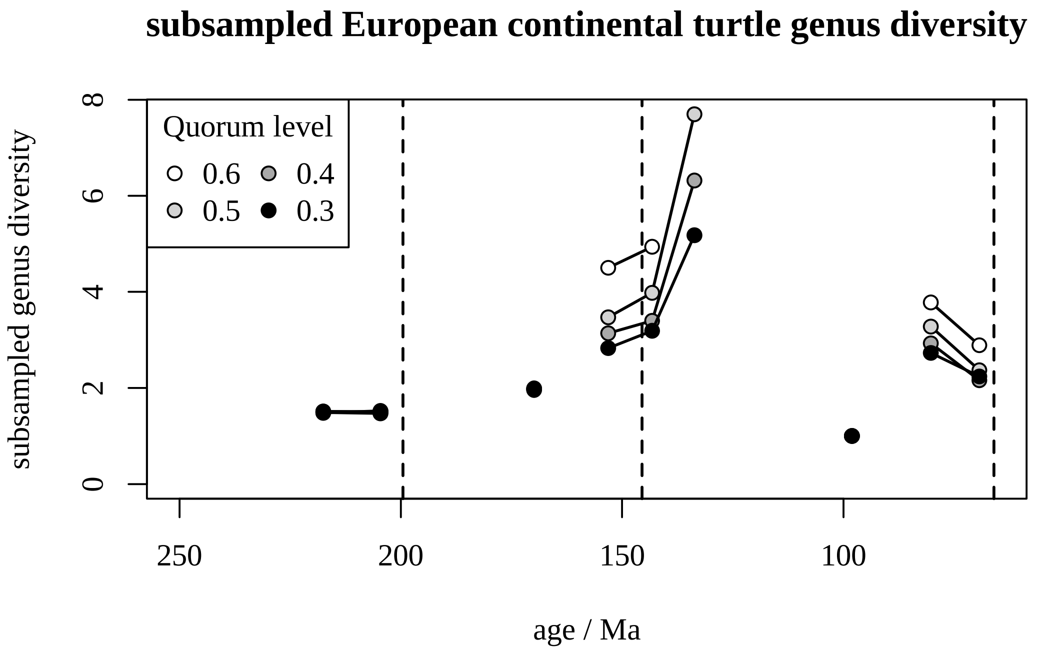

Subsampled European non-marine chelonian genus diversity
Data shown at four quorum levels.

Supplementary Figure 5

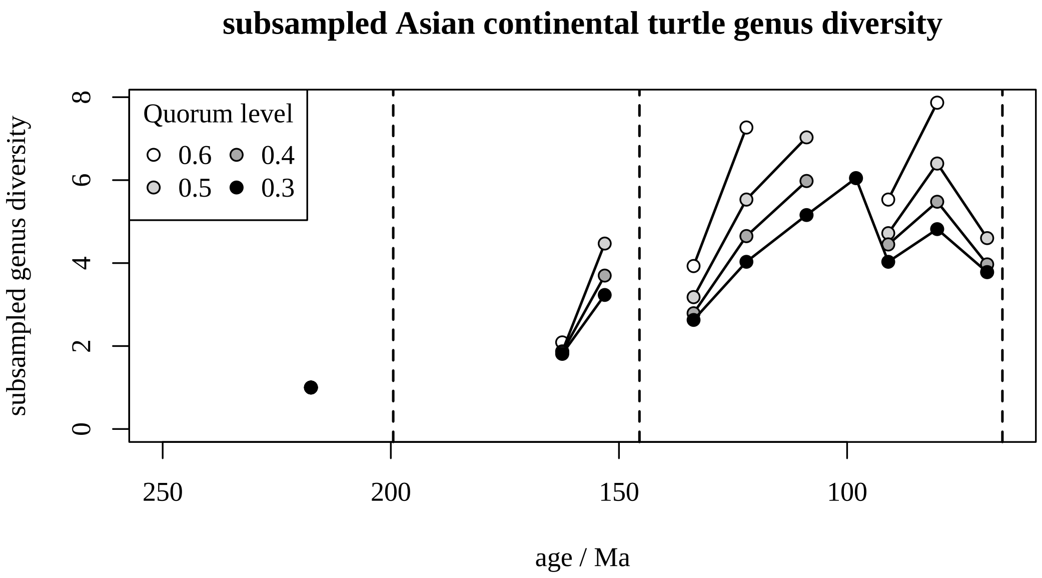

Subsampled Asian non-marine chelonian genus diversity
Data shown at four quorum levels.

Supplementary Figure 6

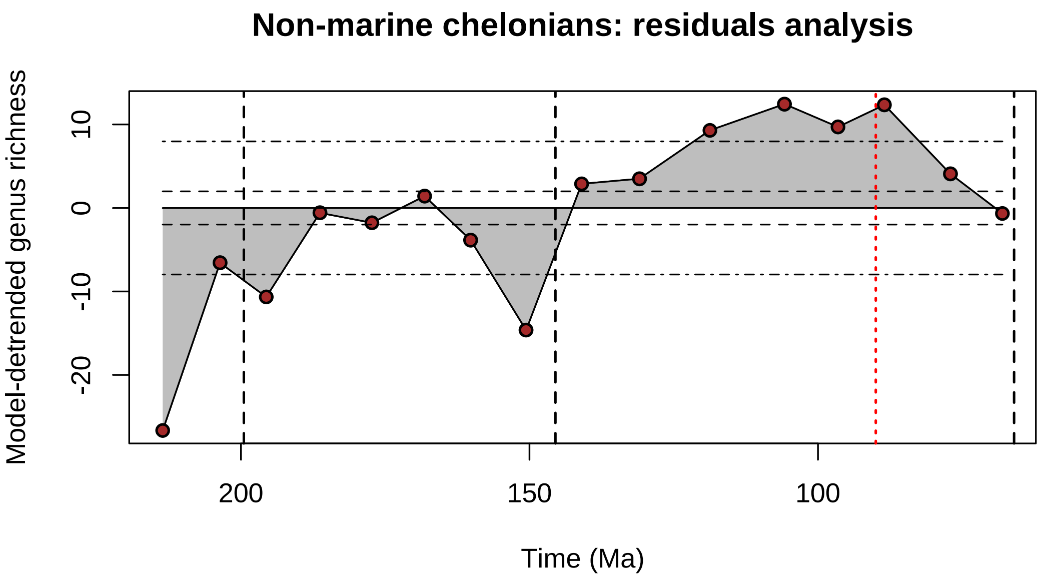

Residual non-marine chelonian genus richness
Non-marine chelonian genus richness after detrending against a model based on the expected diversity predicted from the number of non-marine tetrapod-bearing Paleobiology Database collections per bin. Data available in Table 2.

Supplementary Figure 7

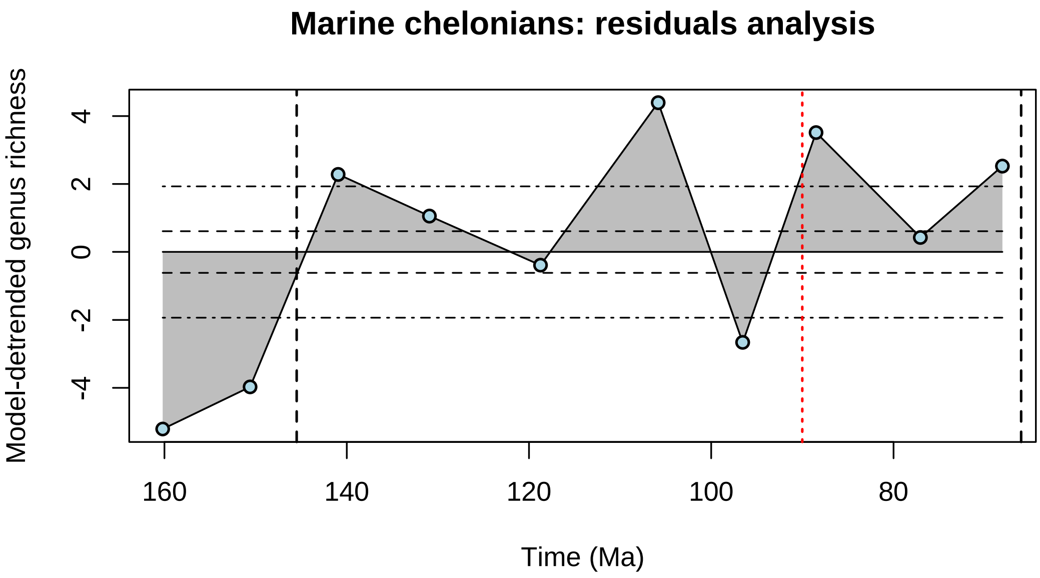

Residual marine chelonian genus richness
Marine chelonian genus richness after detrending against a model based on the expected diversity predicted from the number of marine tetrapod-bearing Paleobiology Database collections per bin. Data available in Supplementary Table 2.

Supplementary Figure 8

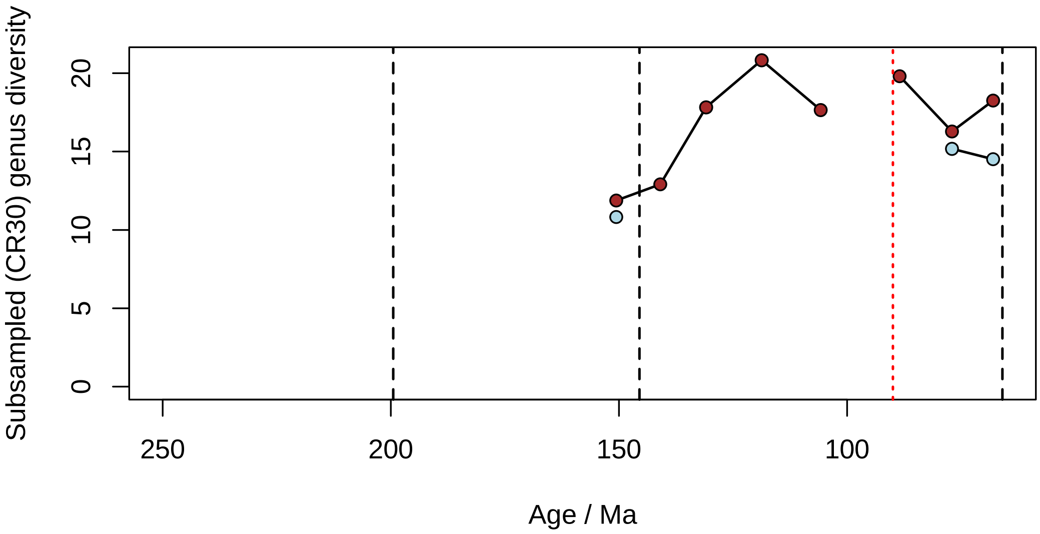

Classical Rarefaction subsample of global chelonian genus richness
Marine (blue) and non-marine (brown) chelonian genera, with an occurrence sampling quota of 30 (CR).

Supplementary Figure 9

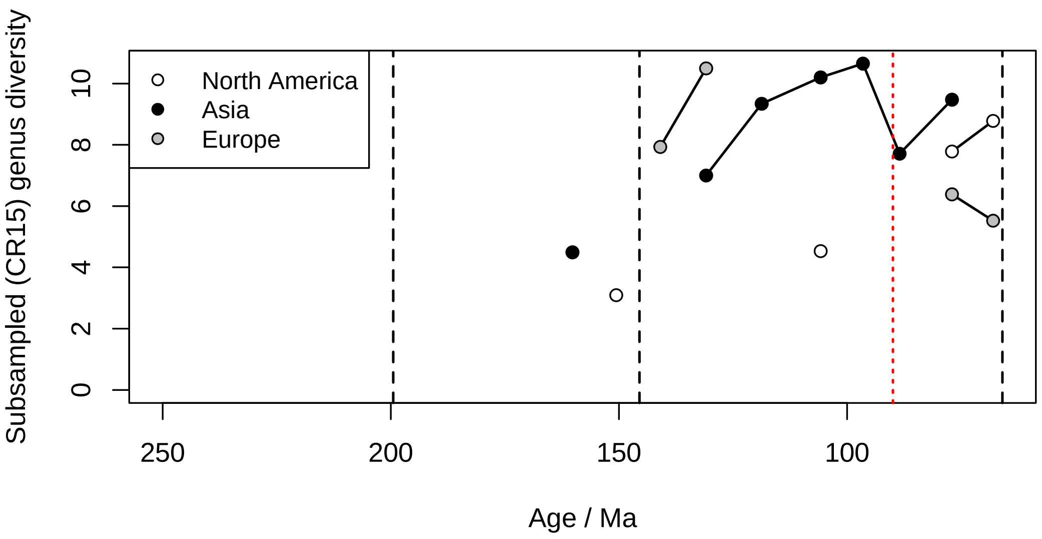

Classical Rarefaction subsample of North American, European and Asian non-marine chelonian genus diversity
Subsampled with an occurrence sampling quota of 15 (CR).

Supplementary Figure 10

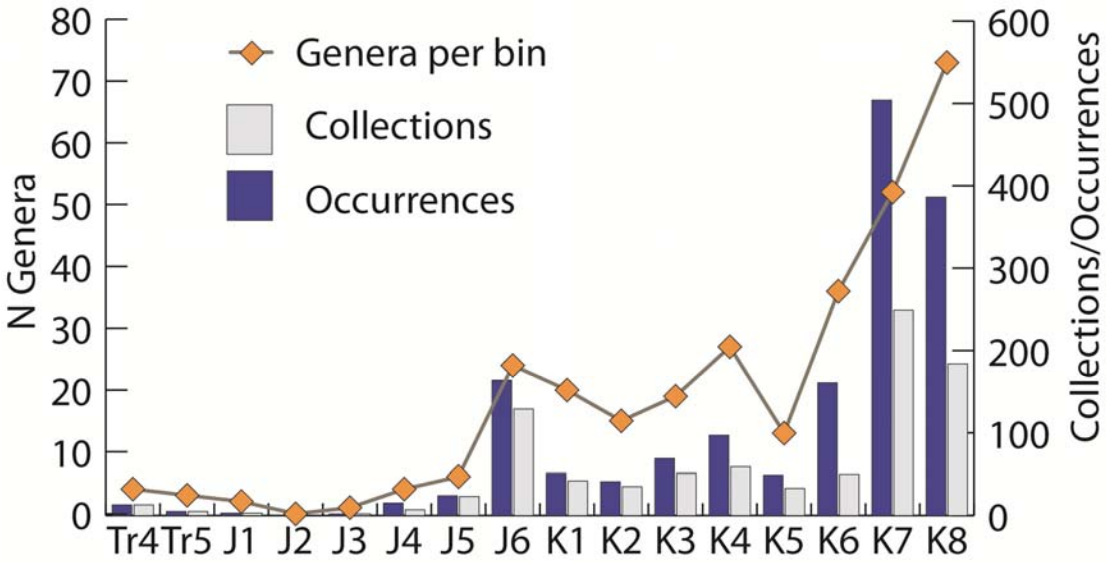

Observed counts of chelonian genera and occurrences per time bin
Yellow diamonds = number of observed genera; Blue bars = number of observed chelonian genera occurrences; Grey bars = number of turtle-bearing PBDB collections. Observed counts after SQS binning protocol.

Supplementary Figure 11

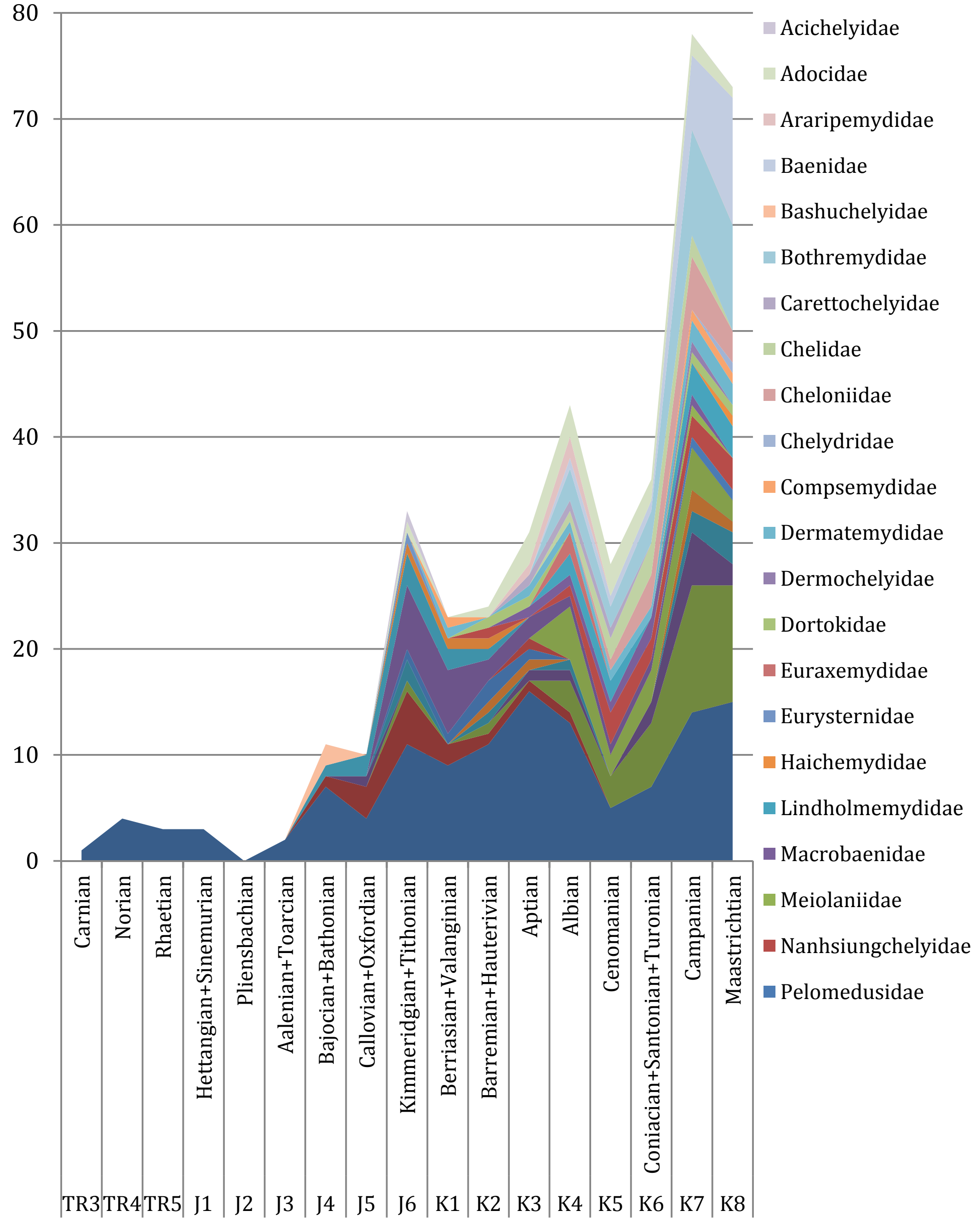

Contribution of different family-level clades of chelonians to Mesozoic genus richness

Genera included in family based on the taxonomic hierarchy as recorded in the Paleobiology Database at time of data download; some genera included in "unassigned to family" have been previously been assigned to family. Data available in Supplementary Table 3.

Supplementary Figure 12

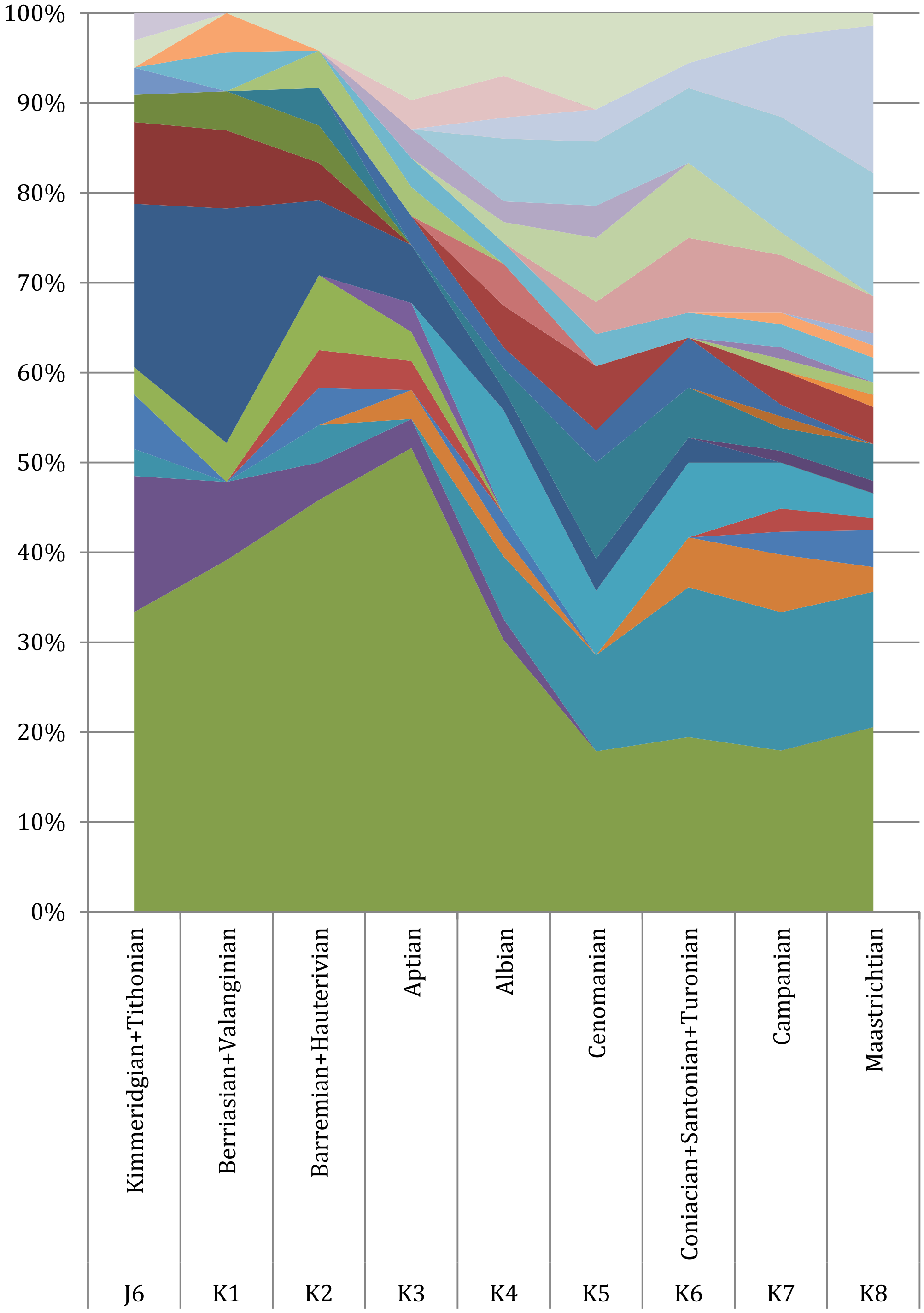

Proportional contribution of different family-level clades of chelonians to genus richness

Genera included in family based on the taxonomic hierarchy as recorded in the Paleobiology Database at time of data download; some genera included in "unassigned to family" have been previously been assigned to family. Legend as for Supplementary Figure 11. Data available in Supplementary Table 3.

Supplementary Table 1

Africa	Angola, Egypt, Jordan, Lesotho, Malawi, Morocco, Niger, Palestinian Territory, Saudi Arabia, South Africa, Syria, Tunisia
North America	Canada, Cuba, Greenland, Mexico, United States
South America	Argentina, Bolivia, Brazil, Chile, Colombia
Asia	China, Japan, Asian Kazakhstan, Kyrgyzstan, Laos, Mongolia, Asian Russia, South Korea, Tajikistan, Thailand, Uzbekistan
Europe	Austria, Belgium, France, Germany, Hungary, European Kazakhstan, Netherlands, Poland, Portugal, Romania, European Russia, Slovenia, Spain, Sweden, Switzerland, United Kingdom

Countries with Mesozoic chelonian occurrences, split by continent for use in Figure 2

Supplementary Table 2.

9 my bin	TR4	TR5	J1	J2	J3	J4	J5	J6	K1	K2	K3	K4	K5	K6	K7	K8
stages	Norian	Rhaetian	Hettangian+Sinemurian	Pliensbachian	Aalenian+Toarcian	Bajocian+Bathonian	Callovian+Oxfordian	Kimmeridgian+Tithonian	Berriasian+Valanginian	Barremian+Hauterivian	Aptian	Albian	Cenomanian	Coniacian+Santonian+Turonian	Campanian	Maastrichtian
Non-marine tetrapod-bearing PBDB collections	1049	212	329	13	89	186	220	1226	313	450	599	452	379	455	2998	3237
Non-marine chelonian raw genus counts	4	2	2	0	2	9	5	19	15	20	30	29	24	29	52	48
Marine tetrapod-bearing PBDB collections	NA	NA	NA	NA	NA	NA	166	324	31	17	25	65	102	135	371	261
Marine chelonian raw genus counts	NA	NA	NA	NA	NA	NA	4	14	4	2	1	8	3	11	21	17

Counts of marine and non-marine chelonian-bearing PBDB collections and corresponding raw genus counts (*after* SQS binning protocol) used in residuals analyses (Supplementary Figures 6 and 7).

Supplementary Table 3.

9 my bin	TR3	TR4	TR5	J1	J2	J3	J4	J5	J6	K1	K2	K3	K4	K5	K6	K7	K8
stages	Carnian	Norian	Rhaetian	Hettangian+Sinemurian	Pliensbachian	Aalenian+Toarcian	Bajocian+Bathonian	Callovian+Oxfordian	Kimmeridgian+Tithonian	Berriasian+Valanginian	Barremian+Hauterivian	Aptian	Albian	Cenomanian	Coniacian+Santonian+Turonian	Campanian	Maastrichtian
Acichelyidae									1								
Adocidae									1		1	3	3	3	2	2	1
Araripemydidae												1	2				
Baenidae													1	1	1	7	12
Bashuchelyidae							2										
Bothremydidae													3	2	3	10	10
Carettochelyidae												1	1	1			
Chelidae													1	2	3	2	
Cheloniidae														1	3	5	3
Chelydridae																	1
Compsemydidae										1						1	1
Dermatemydidae										1		1	1	1	1	2	2
Dermochelyidae																1	
Dortokidae											1	1				1	1
Emydidae																1	
Euraxemydidae													2				
Eurysternidae									1								
Haichemydidae																	1
Lindholmemydidae													2	2		3	3
Macrobaenidae												1	1	1	2	1	
Meiolaniidae																1	
Nanhsiungchelyidae											1		1	3	2	2	3
Pelomedusidae																1	1
Peltochelyidae									1	1	1						
Plesiochelyidae							1	2	3	2	1						
Pleurosternidae									6	6	2	2	1	1	1		
Protostegidae													5	2	3	4	2
Sandownidae												1					
Sinemydidae									1	1	2	1					
Solemydidae											1	1				2	1
Testudoolithidae											1		1		1	1	
Thalassemydidae									2		1		1			2	3
Toxochelyidae								1				1	1		2	5	2
Trionychidae									1		1		3	3	6	12	11

Xinjiangchelyidae							1	3	5	2	1	1	1				
unassigned to family	1	4	3	3		2	7	4	11	9	11	16	13	5	7	14	15
Total	1	4	3	3	0	2	11	10	33	23	25	31	44	28	37	80	73

Counts of genera per family per time bin, used to plot Supplementary Figures 11 and 12.
